# Cholecystectomy for acute cholecystitis. How time-critical are the so called “golden 72 hours”? Or better “golden 24 hours” and “silver 25–72 hour”? A case control study

**DOI:** 10.1186/1749-7922-9-60

**Published:** 2014-12-16

**Authors:** Peter Ambe, Sebastian A Weber, Hildegard Christ, Dirk Wassenberg

**Affiliations:** Helios Klinikum Wuppertal, Department of Surgery II, Witten/Herdecke University, Heusner Strasse 40, 42283 Wuppertal, Germany; Department of Internal Medicine, St. Elisabeth Hospital Hohenlind, 50377 Köln, Germany; Department of medical statistics and epidemiology, University of Cologne, Cologne, Germany; Department of General, visceral and thoracic surgery, St. Remigius Hospital Opladen, An St. Remigius 26, 51379 Leverkusen, Germany

**Keywords:** Acute cholecystitis, Laparoscopic cholecystectomy, Early cholecystectomy, Immediate cholecystectomy, Gallbladder inflammation, Tokyo guidelines, Timing of cholecystectomy

## Abstract

**Introduction:**

Early cholecystectomy within 72 hours has been shown to be superior to late or delayed cholecystectomy with regard to outcome and cost of treatment. Recently, immediate cholecystectomy within 24 hours of onset of symptom was proposed as standard procedure for the management of fit patients presenting with acute cholecystitis. We sort to find out if there are any differences in surgical outcomes between patients managed within 24 h and those managed 25-72 h following symptom begin for acute cholecystitis.

**Patients and methods:**

A retrospective analysis was performed. The outcomes of patients undergoing laparoscopic cholecystectomy within 24 h were compared to those of patients managed 25-72 h following symptom onset for acute cholecystitis.

**Results:**

35 patients managed 25-72 h following begin of symptoms were matched with 35 patients with similar baseline features, medical comorbidities and disease severity managed within 24 hours of symptom onset. There were no significant differences in the duration of surgery, postoperative complications, rate of conversion and length of hospital stay.

**Conclusion:**

Immediate laparoscopic cholecystectomy for acute cholecystitis within 24 hour of symptom onset is not superior to surgery 25–72 hour after symptoms begin. Laparoscopic cholecystectomy for acute cholecystitis therefore can be safely performed anytime within the golden 72 h.

## Introduction

Acute cholecystitis (AC) is a common diagnosis in the surgical practice with a clear indication for surgery. Although widely discussed in the past, unequivocal evidence exists supporting the superiority of early laparoscopic cholecystectomy within 72 hours over delayed LC with respect to outcome and cost of treatment [1–8]. This trend was confirmed in a recently published randomized study in patients managed within 24 hours of admission [9]. Cholecystectomy however, may not always be possible within 24 hours of admission for many different reasons. In such cases, surgery should be performed within 72 hours as recommended in several guidelines [10–12]. The aim of this study was to compare the outcomes of patients undergoing LC within 24 h of symptom begin on one hand to those of patients managed 25 to 72 h after symptom begin for AC on the other hand.

## Patients and methods

A retrospective review of the charts of patients undergoing cholecystectomy for AC from January 2009 to December 2013 in the department of surgery of a primary care hospital in Germany was performed. Baseline characteristics including age, sex, body mass index (BMI) and medical comorbidities as defined by the American Society of Anesthesiology (ASA) were retrieved for each patient.

Acute cholecystitis was diagnosed as outlined in the Tokyo guidelines [13, 14]. The diagnosis was confirmed during surgery and following histopathology. Only patients managed within 72 hours of symptom begin were included for analysis. All patients were placed on intravenous antibiotics upon admission which was continued after surgery. Perioperative data including the duration of anesthesia, the duration of surgery, conversion to open surgery, postoperative complications and the length of postoperative hospital stay were retrieved from surgical documentation sheets, surgeon’s notes and discharge records.

All surgeries in this study were performed by experienced attending surgeons. Laparoscopic cholecystectomy [15] was carried out using four incisions with pneumoperitoneum installed via a sub-umbilical mini-laparotomy with the maximum intraabdominal pressure kept at 12 mmHg.

The data collected was analyzed using the Statistical Package for Social Science (SPSS®), IBM, version 22. The study population was statistically described using absolute case numbers, percentages, medians and interquartile ranges. Significances were calculated using the Fisher' s exact test with levels of significance set at p < 0.05.

Patients operated upon 25 to 72 h after symptom begin (study group) were matched with regard to baseline and clinical features (same gender, similar ages, disease severity, BMI, ASA and APACHE II Scores) as well as disease severity grade (as outlined in the Tokyo guidelines) with patients operated upon within 24 h following symptom begin (control group). Both groups were comparable with regard to demographic and clinical characteristics.

Primary endpoints included the duration of anesthesia, the duration of surgery and postoperative complications. Secondary endpoints included the length postoperative of hospital stay and hospital mortality.

## Results

**Figure 1:**
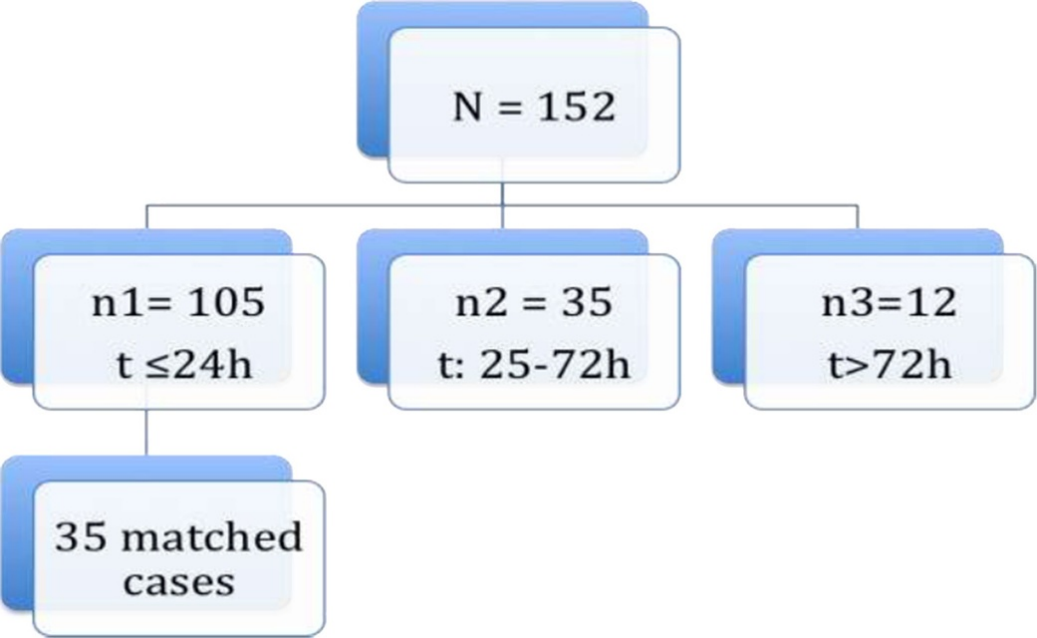
**Distribution of the study population.** 35 Patients undergoing surgery 25-72 h following symptom begin were compared to 35 selected patients with similar demographic and clinical parameter from the groups managed within 24 h of symptom onset.

Within the period of investigation 152 cases of AC were managed surgically. The distribution of study population is represented in Figure 1. Thirty-five patients were managed within 25-72 h following symptom begin (study group). Thirty-five patients with similar characteristics to those of the study group were selected from the 105 patients managed within 24 h of symptom begin, Table 1. The demographic characteristics of the study population are summarized in Table 2. Both groups were comparable in all cases.

**Table 1:** **Patients with similar baseline and clinical characteristics were selected from the group managed within 24 h and matched with those managed 25-72 h**

Case number	Gender		Age/yrs		ASA		BMI		Severity grade		APACHE II score	
	≤24 h	25- 72 h	≤24 h	25- 72 h	≤24 h	25- 72 h	≤24 h	25- 72 h	≤24 h	25- 72 h	≤24 h	25- 72 h
1	M	M	69	68	2	2	32.4	23.8	1	1	5	7
2	M	M	74	74	3	3	35.2	26.1	2	3	9	7
3	F	F	69	70	3	3	32.4	35.8	2	2	8	6
4	M	M	82	84	4	3	28.7	27.4	3	3	18	17
5	F	F	30	22	2	2	25.5	25.4	2	2	1	1
6	F	F	68	61	1	2	26.2	41.5	2	2	6	5
7	F	F	39	39	2	2	32.3	24.8	1	1	2	1
8	M	M	76	74	4	4	34.9	33.1	3	3	18	19
9	M	M	74	74	3	4	25.1	26.8	2	3	17	18
10	M	M	80	76	2	2	28.4	23.6	2	1	14	13
11	F	F	46	45	2	1	38.6	28.7	1	1	3	2
12	M	M	42	43	2	2	28.1	27.0	1	1	2	2
13	F	F	37	37	2	1	26.3	29.7	1	1	1	1
14	F	F	60	55	2	2	32.8	31.0	1	1	4	4
15	F	F	84	81	2	1	25.4	20.8	2	1	11	14
16	M	M	76	71	3	3	31.6	25.6	1	1	11	11
17	M	M	57	60	2	2	29.6	29.0	1	1	5	8
18	F	F	45	47	2	2	31.3	33.2	1	1	3	2
19	M	M	34	31	1	1	26.2	17.0	1	1	1	1
20	F	F	43	42	3	3	29.4	26.6	1	1	2	3
21	F	F	76	71	3	3	27.2	31.6	2	1	9	6
22	M	M	56	56	2	1	24.2	24.7	1	1	6	6
23	M	M	43	45	1	2	24.2	22.0	1	1	1	2
24	M	M	80	79	3	3	16.2	19.4	1	2	9	9
25	F	F	51	54	2	2	24.8	24.4	1	1	4	3
26	M	M	52	59	2	2	28.7	26.2	1	1	4	4
27	M	M	73	74	2	2	30.8	29.2	2	1	8	5
28	F	F	61	65	3	3	26.0	33.5	3	3	15	13
29	F	F	82	83	3	3	31.2	21.5	1	1	7	5
30	M	M	65	61	2	1	25.7	26.5	1	1	8	5
31	M	M	64	63	2	2	25.1	21.6	1	2	9	7
32	M	M	79	77	3	3	33.9	31.2	3	3	13	11
33	M	M	82	87	3	3	29.9	27.6	2	3	19	18
34	M	M	71	76	2	2	30.2	33.8	1	1	11	11
35	F	F	68	63	2	1	30.2	34.1	1	1	11	11

Both groups were comparable in terms of demographic and clinical parameters.

**Table 2:** **Summary of the baseline characteristics of the cohort**

Feature		≤24 h	25 - 72 h	P-value
Gender (F/M)		15/20	15/20	/
Median age (interquartil range)		68.0 (30.0)	63.0 (27.0)	0.32
Median BMI (interquartil range)		28.7 (5.9)	26.8 (6.8)	0. 19
ASA	1-2	22	22	/
	3-4	13	13	

Both groups were comparable with regard to demographic characteristics.

There was no significant difference in the duration of anesthesia, the duration of surgery and the length of postoperative hospital stay amongst both groups. Five cases (14.2%) were converted to open cholecystectomy in the group managed within 24 h, while 3 cases (8.6%) were converted in the group operated upon within 25-72 h of symptom onset. This difference was not statistically significant, p = 0.23.

Two complications, including one patient with pneumonia and one with wound infection, were recorded in the group operated upon within 24 hours (5.7%). Five complications, including three patients with bile leak, one patient with wound infection and one patient with acute renal failure, were recorded in the group managed between 25-72 h of symptom begin (14.2%). This difference was not statistically significant (p = 0.42), Table 3. There was no mortality in both groups.

**Table 3:** **Summary of the perioperative data**

Parameters	≤24 h	25-72 h	P-value
Median duration of anaesthesiology (interquartile range)	120.0 (45.0) min	115.0 (35.0) min	0.82
Median duration of surgery (interquartile range)	70.0 (35.0) min	65.0 (30.0) min	0.23
Rate of complication	5.7%	14.2%	0.42
Median duration of postoperative stay (interquartile range)	7.0 (3.0) min	6.0 (2.0)	0.65

Min: minutes.

## Discussion

The optimal timing of surgery for patients with AC has been a topic of controversy in the past. Initially, patients were managed conservatively with the aim of “cooling down” the inflammation, and then perform cholecystectomy weeks later. The heterogeneity of patients suffering from AC and their medical co-morbidities it difficult to standardize treatment [16]. Acute cholecystitis was once considered a relative contraindication for LC at the beginning of the laparoscopic era, mainly due to high rates of complications and conversion. This trend however changed following growing expertise in laparoscopy. Nowadays, laparoscopic cholecystectomy is the gold standard for the management of benign gallbladder disorders and belongs to one of the most commonly performed procedures in surgery [17, 18]. Current data suggest that early LC for acute cholecystitis is superior to late or delayed LC with regard to outcome and cost of treatment [2, 19].

The term „early“ is rather vaguely defined in the literature [20, 21]. In some series, „early“ defines the begin of symptoms while the same term is used with regard to the time of admission in other series. In this study, “early” was defined with respect to symptom begin. Generally speaking, early cholecystectomy is performed within a time interval of 72 h, the so called golden 72 h [22].

In a recently published multi-center randomized study by Gutt et. al., laparoscopic cholecystectomy performed within 24 h of admission was shown to be superior to delayed LC with regard to outcome. The authors concluded that immediate LC should become the treatment of choice for operable patients with AC [9]. This conclusion however, cannot be generally applied at all levels of patient care. Furthermore, immediate LC may not always be possible for different reasons. A number of patients presenting with AC may require special consultations and correction of co-morbidities (e.g. those on oral anticoagulation treatment) before undergoing surgery. Besides, experienced laparoscopic surgeons may not be available within 24 h, as may be the case in quiet a number of primary care hospitals. In such cases, LC should be performed within 72 h.

The aim of this study was to compare the outcomes of patients with AC managed within 24 h of symptom begin to those of patients managed 25-72 h following symptom onset. Data of patients undergoing LC in a primary care hospital in Germany was retrospectively analyzed. Thirty-five patients with AC managed within 24 h were matched (similar baseline characteristics, comorbidities and disease severity) with 35 patients managed 25 - 72 h after symptom onset. All surgeries were performed laparoscopically by surgical attendings with expertise in laparoscopy.

Surgery for acute cholecystitis could be time critical. According to Zhu et. al., gallbladder inflammation during the first 72 h of onset of symptoms may not involve structures within the triangle of Calot [23]. Surgical dissection within this critical period therefore appears easiest due to lack of organized adhesions. Cholecystectomy within this time frame reduces the risk of injury to the structures within the triangle of Calot. This is reflected in the low rates of complication and conversation.

There was no significant difference amongst both groups with respect to the duration of anesthesia and the duration of surgery. Equally, there was no significant difference in the rates of conversion and morbidity between both groups. All cases of conversion were due to the inability to clearly identify the structures within the space of calot.

Interestingly, three cases of bile leak were recorded in the study group. These complications occurred in patients with severity grade III and histopathologic evidence of necrotizing cholecystitis.

We could not prove any difference in outcome between the group managed within 24 h and that managed 25 - 72 h of onset of symptoms. Our results therefore suggest that it is not necessary to perform LC for AC within 24 h following symptom onset.

Taken together, a division of the critical time frame, i.e. the so called “golden 72 h” for the surgical management of acute cholecystitis into a more favorable “golden 24 h” and a less favorable “silver 25-72 h” could not be justified in this series.

This study is limited by the relatively small size of the cohorts and the retrospective study design. Since the study did not include consecutive patients, there might have been some degree of bias in the selection of the patients for the matching group. Therefore the trend shown in this study must be validated in prospective studies with larger case numbers.

## Conclusion

Laparoscopic cholecystectomy for acute cholecystitis must not be performed within 24 h of admission. The golden 72 h time frame however should be maintained where possible.
